# Materials, Designs, and Implementations of Wearable Antennas and Circuits for Biomedical Applications: A Review

**DOI:** 10.3390/mi15010026

**Published:** 2023-12-22

**Authors:** Minye Yang, Zhilu Ye, Yichong Ren, Mohamed Farhat, Pai-Yen Chen

**Affiliations:** 1 State Key Laboratory for Manufacturing Systems Engineering, Electronic Materials Research Laboratory, Key Laboratory of the Ministry of Education, Engineering Research Center of Spin Quantum Sensor Chips, Universities of Shaanxi Province, School of Electronic Science and Engineering, Xi’an Jiaotong University, Xi’an 710049, China; 2 Department of Electrical and Computer Engineering, University of Illinois Chicago, Chicago, IL 60607, USA; zhiluye@xjtu.edu.cn (Z.Y.); yren27@uic.edu (Y.R.); pychen@uic.edu (P.-Y.C.); 3 State Key Laboratory for Manufacturing Systems Engineering, The Key Laboratory of Biomedical Information Engineering of Ministry of Education, Center for Mitochondrial Biology and Medicine, School of Life Science and Technology, International Joint Laboratory for Micro/Nano Manufacturing and Measurement Technology, Xi’an Key Laboratory for Biomedical Testing and High-end Equipment, Xi’an Jiaotong University, Xi’an 710049, China; 4 Division of Computer, Electrical and Mathematical Sciences and Engineering, King Abdullah University of Science and Technology (KAUST), Thuwal 23955-6900, Saudi Arabia; mohamed.farhat@kaust.edu.sa

**Keywords:** flexible/soft antennas, wearable electronics, flexible conductors, biomedical sensing, epidermal sensors, implantable sensors

## Abstract

The intersection of biomedicine and radio frequency (RF) engineering has fundamentally transformed self-health monitoring by leveraging soft and wearable electronic devices. This paradigm shift presents a critical challenge, requiring these devices and systems to possess exceptional flexibility, biocompatibility, and functionality. To meet these requirements, traditional electronic systems, such as sensors and antennas made from rigid and bulky materials, must be adapted through material science and schematic design. Notably, in recent years, extensive research efforts have focused on this field, and this review article will concentrate on recent advancements. We will explore the traditional/emerging materials for highly flexible and electrically efficient wearable electronics, followed by systematic designs for improved functionality and performance. Additionally, we will briefly overview several remarkable applications of wearable electronics in biomedical sensing. Finally, we provide an outlook on potential future directions in this developing area.

## 1. Introduction

The interdisciplinary confluence of biomedicine and engineering has led to a transformative era in contemporary lifestyles, notably within the realm of self-health monitoring [1]. Traditionally, the clinical assessment of chronic ailments, inflammatory conditions, and hemorrhagic disorders has predominantly relied upon subjective evaluations by healthcare practitioners, thereby manifesting inherent limitations in terms of precision and real-time feedback. Conversely, the diagnosis of critical diseases and injuries, such as traumatic brain injuries [2,3,4], intracranial hemorrhages [5,6,7], and gastric ulcers [8,9], has disproportionately hinged on the deployment of catheter-based apparatuses, which often entail substantial heavy-duty equipment and high cost. Recently, the advent of wearable electronic devices featuring soft and pliable antennas [10,11,12] and functional circuitry [13,14,15] has presented an alternative avenue for clinical diagnostics and post-surgery therapeutic protocols, characterized by the wireless, continuous, and instantaneous monitoring of human physiological and vital signs.

These wearable electronic devices, in sharp contrast to their traditional bulky and rigid counterparts, have been meticulously engineered to embody the merits of lightness [16], compactness [17], and unobtrusiveness [18], thereby facilitating their fabrication and measurement convenience. It is worth noting that these devices, as denoted by the term “wearable”, are constructed from intrinsically flexible and soft materials [19,20,21]. A salient illustration involves the adoption of silver nanowires (AgNWs) [22,23,24] as a replacement for conventional copper as the conductive material, where the intertwining of AgNWs, spanning hundreds of micrometers in length and tens of nanometers in radius, provides the desired electrical conductivity. Nonetheless, the practical limitations pertaining to their deployment still persist, an issue that we will discuss later. Furthermore, the flexibility and pliability intrinsic to wearable devices are chiefly achieved by substituting bulky and rigid substrates, such as FR-4 and RT/duroid 5880, with some soft materials. Recent research endeavors have been directed towards the utilization of polymer composite materials, exemplified by porous styrene–ethylene–butylene–styrene (SEBS) [25,26,27], polyurethane (PU) [28,29,30], and polydimethylsiloxane (PDMS) [31,32,33] as substrates for wearable devices, owing to their superior biocompatibility and remarkable flexibility.

Taking into account the emerging materials constituting the foundation of wearable electronics, it is commonly observed that they may not exhibit the same level of electrical properties in comparison to traditional materials such as copper and FR-4. Whether it be the diminished conductivity of the conductive materials or the increased dielectric losses of the substrate materials, sophisticated design modifications of the system are often necessitated to preserve the functionality of wearable antennas and circuits. In addition to these challenges, wearable antennas and circuits may operate in close proximity to human skin, or even be embedded within the human body, a circumstance that can detrimentally affect their performance [34]. For instance, the human body, being an absorber of electromagnetic energy, can significantly influence the operation frequencies and gain of antennas. Consequently, in this scenario, a certain specification of the wearable antenna may be mandatorily required to maintain the stable operation status of antennas. Despite the lesser impact of the human body on the performance of wearable circuits, direct contact with the epidermis and/or implantation within the body places a premium on the devices’ exceptional biocompatibility. However, materials endowed with exceptional biocompatibility may not invariably possess remarkable electrical properties, thereby necessitating novel circuit designs to optimize system efficacy.

Notwithstanding those challenges encountered, the ever-increasing attention devoted to self-health monitoring, especially in the aftermath of the global COVID-19 pandemic, has propelled the advancement of this interdisciplinary domain. Benefiting from the rapid evolution of innovative, flexible materials [35,36] and the application of unique electronic device designs [37,38,39], wireless wearable sensing systems have registered noteworthy accomplishments in diverse domains, including respiration monitoring [40,41], electrocardiogram (ECG) monitoring [42,43,44], intracranial pressure assessment [45,46,47,48,49], etc. It is natural to say that the practical implementation of these wearable systems has the potential to prompt progress within clinical medicine and life health management. In this comprehensive review article, our primary focus will be directed toward the traditional/emerging materials employed in the construction of wearable electronics, alongside distinctive system designs tailored for performance enhancement and specialized functionalities. Additionally, we will explore several prominent biomedical applications underpinned by wearable electronics and provide a forward-looking perspective on this nascent field.

## 2. Traditional/Emerging Materials for Wearable Antennas and Circuits

**Figure 1:**
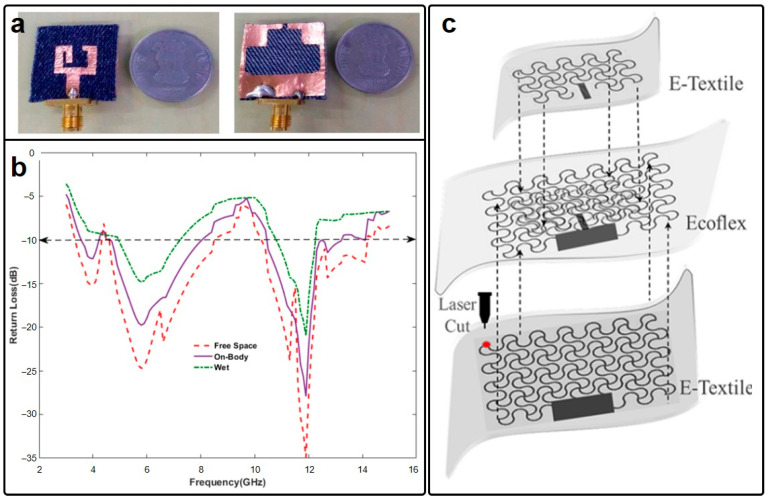
(**a**) Front and back views of the jeans-based wearable antenna and its operation frequency in (**b**). (**c**) Structure of the wearable antenna based on Ecoflex and silver-plated knitted textile. (**a**–**c**) are reprinted from open-access works [65,66].

Copper, renowned for its exceptional electrical conductivity and abundant natural storage, has enjoyed extensive utilization as the preeminent conductive material for centuries. During the embryonic stages of wearable electronics development, it was customary for researchers to persist with copper as the conductive material, which comes at the cost of flexibility. A notable exemplar of this paradigm is evident in textile-based wearable antennas [50,51], which typically employ fabrics, including denim [52,53], cotton [54,55], and flannel [56,57,58], as substrates. Such antennas, often referred to as intelligent garments [59], e-textiles [60], or smart fabrics [61], incorporate a thin copper-based conductive layer or conductive fabrics [62,63,64] onto these supple fabrics, thereby enabling the integration of wearable electronic functionality. A pertinent illustration is elucidated in Ref. [65] by Ashok Yadav et al., wherein a flexible antenna designed for the C and X/Ku bands is presented in Figure 1a,b. The compact dimensions of this antenna indeed complement the rigidity of the copper layer, rendering the entire device suitable for integration into clothing. Further, the inclusion of a defected ground structure mitigates frequency shifts when worn on the body, ensuring a relatively stable performance. However, the inherent rigidity of the copper ground layer may still impose discomfort on the wearer. Fatemeh Nikbakhtnasrabad and collaborators have additionally advanced a fully flexible antenna configuration [66], where both the radiated patch and ground plane are fabricated using conductive fabrics (Figure 1c). The conductive layer consists of silver-plated knitted textiles, which exhibit excellent compatibility with the fabrics and maintain a commendable electrical conductivity of 1.14 × 10^3^ S/m—sufficient to sustain antenna operation.

**Figure 2:**
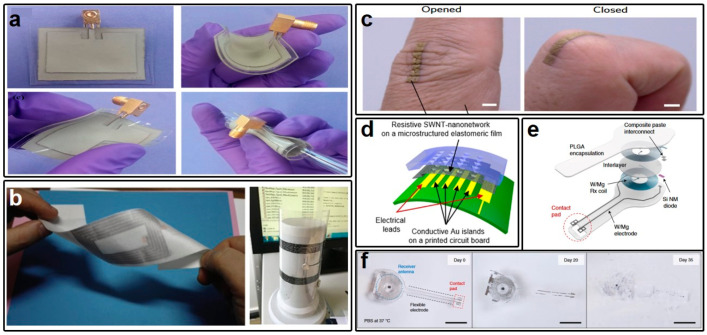
(**a**) Picture of the wearable antenna based on PDMS substrate with AgNW/PDMS composite as conductive material and its bending view. (**b**) Wearable antenna based on graphene multilayer fabricated on silk and PET for NFC application, respectively. (**c**) Picture of the epidermis wearable strain sensor directly fabricated on the human skin. (**d**) Diagram of the wearable plantar pressure sensor based on the Au-SWNT nanonetwork. (**e**) Structure of a fully bioresorbable implantable pacemaker and its bioresorption process in (**f**). (**a**) is reprinted with permission from Ref. [69], Copyright 2014 American Chemical Society. (**b**) is reprinted from open-access literature [74]. (**c**) is reprinted with permission from Ref. [82], Copyright 2017 Springer Nature. (**d**) is reprinted with permission from Ref. [83], Copyright 2019 American Chemical Society. (**e**,**f**) are reprinted with permission from Ref. [84], Copyright 2021 Springer Nature.

In addition to conductive textiles, emerging nanomaterial-based wearable antennas have garnered attention for their potential in diverse implementation scenarios. Unlike textile-based wearable antennas, which are primarily integrated into clothing, nanomaterial-based alternatives, often referred to as e-skins [67,68], are specifically designed for direct contact with the human epidermis. Lingnan Song et al. introduced a AgNWs/PDMS composite material-based wearable antenna (Figure 2a) characterized by superior electrical conductivity (8.13 × 10^5^ S/m) [69]. This composite material offers fewer implementation constraints, as it can be spray-printed onto various soft substrates. Furthermore, the AgNW-based conductive layer exhibits excellent biocompatibility. Besides the AgNW-based conductive materials, graphene has also gained prominence as a conductive material for wearable antennas due to its exceptional thermal [70,71] and electrical characteristics [72,73], coupled with outstanding flexibility. A notable instance, presented in Ref. [74] by A. Scida and colleagues (Figure 2b), features a conductive layer composed of stacked graphene multilayers with a remarkable conductivity of 4.20 × 10^5^ S/m—significantly surpassing the previously discussed materials. The ease of implementation associated with graphene conductive material makes it a promising candidate for wearable electronics, as it can be readily fabricated on diverse substrates. As exemplified in Figure 2b, a wearable near-field communication (NFC) antenna based on graphene multilayers is fabricated on silk and polyethylene terephthalate (PET), respectively. Furthermore, ongoing advancements in the utilization of carbon nanotubes (CNT) [75,76], MoS_2_ [77,78,79], hydrogel [80,81], and other innovative materials as conductors for wearable antennas have signified the blossoming exploration of diverse options in this domain.

In the context of wearable electronic systems, the stringent requirements for electrical conductivity in wearable antennas differ markedly from the considerations surrounding the use of conductive materials in wearable sensors, where biocompatibility takes precedence over extreme conductivity. Akihito Miyamoto et al. [82] have proposed the utilization of nanomeshed gold (Au) as a conductor in on-skin sensors, as depicted in Figure 2c. This conductor offers exceptional biocompatibility, enabling direct contact with human skin while simultaneously offering a remarkable conductivity of up to 1.9 × 10^6^ S/m, surpassing the previously discussed emerging materials. The authors have conducted tests on a 2.5 mm-wide, 12 mm-long conductor, revealing minimal degradation in conductance, from 2.9 × 10^−3^ S to 7.1 × 10^−4^ S, even after undergoing 500 stretching cycles at a 25% strain—demonstrating promising potential for utilization as a strain sensor. In a separate development, Hochan Chang et al. introduced a novel piezoresistive sensor design utilizing single-walled carbon nanotubes (SWNT) to bridge the Au conductor (Figure 2d) [83]. This sensor effectively monitors pressure variations by detecting changes in resistance across the entire device. In addition to sensing applications, implantable medical devices, such as pacemakers positioned in proximity to the heart, impose even more stringent requirements for biocompatibility. As reported in Ref. [84] by John A. Rogers, tungsten-coated magnesium (W/Mg) has been employed as the conductive material fabricated upon the substrate of poly(lactide-co-glycolide) (PLGA) in such implantable pacemakers, as shown in Figure 2e. This conductor not only exhibits outstanding conductivity within the designated operation period, but also possesses the remarkable attribute of bioresorbability. After the designated period, the conductor can be entirely absorbed by surrounding biofluids and subsequently excreted from the body via natural biochemical and metabolic processes (Figure 2f).

While we have highlighted a select few nanomaterial-based conductors, it is noteworthy that copper remains the predominant material in wearable circuits, especially in the context of epidermal sensors. This preference arises from the compatibility of copper with the fabrication processes of flexible printed circuit boards (FPCBs), particularly in applications necessitating complicated functionalities. Nevertheless, it is imperative to acknowledge that although labeled as “flexible,” such devices exhibit limited bendability and are incapable of tolerating twisting, stretching, or kneading, thereby restricting their range of implementation scenarios.

**Figure 3:**
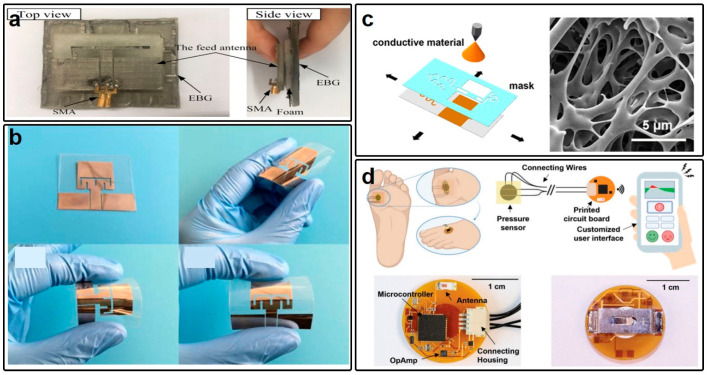
(**a**) Top view and bending view of the wearable antenna fabricated upon PDMS substrate and conductive fabrics. (**b**) Pictures of the wearable antenna using silver nanoparticles and PET substrate showing its flexibility. (**c**) Structure of the porous nanomaterial-based smart facemask and the microscopic image of the porous structure. (**d**) Diagram of a wireless wound healing system and the structure of the wearable sensor based on PI and copper. (**a**) is reprinted with permission from Ref. [91], Copyright 2019 John Wiley & Sons. (**b**) is reprinted with permission from Ref. [92], Copyright 2016 John Wiley & Sons. (**c**) is reprinted with permission from Ref. [95], Copyright 2022 American Chemical Society. (**d**) is reprinted with permission from Ref. [96], Copyright 2012 John Wiley & Sons.

Whereas the electrical characteristics of a wearable electronic system predominantly hinge upon the conductive materials employed, the flexibility and softness are primarily attributed to the properties of the substrates. In wearable antennas, substrates effectively insulate the antennas from direct contact with the epidermis, thereby minimizing the influence of the human body on antenna performance. Conversely, in wearable circuits, substrates are not imperative for functional maintenance but serve to enhance system stability and mitigate potential risks associated with poorly biocompatible conductors. In the domain of wearable antennas, fabrics have emerged as viable candidates to provide adequate antenna metrics. To realize wearable antennas designed for direct attachment to human skin, various polymer materials, including PDMS [31], PET [85,86], PU [87,88], polyimide (PI) [89], and Ecoflex [90], have been explored as substrates. PDMS, noted for its low fabrication cost and high viscosity, rendering it compatible with many emerging conductive materials requiring inkjet-printing or spray-painting techniques, has found widespread use in wearable electronics. Guoping Gao et al. employ PDMS as the substrate for the wearable antenna with an electromagnetic bandgap (EBG) structure shown in Figure 3a, facilitating the operation covering 2.3 GHz to 2.6 GHz for the industrial, scientific, and medical (ISM) band [91]. The participation of the EBG structure indeed increases the performance of this wearable antenna in terms of the reflection coefficient, gain, and radiation efficiency. Further, the operation status of this PDMS-based wearable antenna may be rather robust when subject to bending manipulations and operating close to the human body. However, high viscosity, while advantageous in some respects, may readily accumulate dust particles, complicating the fabrication process. In this regard, PET emerges as a suitable alternative to PDMS. Caixia Liu and her group propose the use of PET substrate in combination with nano-silver ink to establish a coplanar waveguide (CPW)-fed antenna (Figure 3b) [92]. This device, characterized by full transparency and flexibility, maintains a pristine surface devoid of adhered dust particles.

Another class of flexible substrate that has attracted significant research attention in recent years is styrene–ethylene–butylene–styrene (SEBS) [93,94]. Traditional SEBS, however, is unsuitable for wearable applications due to its poor air permeability. Zhilu Ye and her collaborators have proposed a phase-separation method for the preparation of porous SEBS (Figure 3c) [95]. The resulting porous SEBS substrate exhibits excellent air permeability and passive cooling characteristics, offering heightened wearer comfort compared to PDMS, PET, and PU-based devices. Furthermore, SEBS demonstrates superior flexibility and provides robust support for electronic systems. Wearable antennas and circuits can be readily fabricated on SEBS substrates, under-scoring their versatility and potential in this domain.

In addition to the representative flexible substrates commonly employed in wearable antennas, PI stands out as another primary substrate material for FPCB-based wearable circuits. PI offers exceptional thermal properties that surpass those of previously mentioned substrates, withstanding temperatures exceeding 400 °C, simplifying the integration of electronic components through soldering processes. Therefore, the PI substrate, as depicted in Figure 3d, demonstrates the capacity to robustly support large-scale and complicated electronic systems, accommodating multifaceted functionalities, particularly in the realm of multimodal sensing; for example, the smart bandages necessitating sophisticated electronic systems for the plantar pressure monitoring, as reported in Ref. [96] by Xueju Sophie Wang and her group.

**Figure 4:**
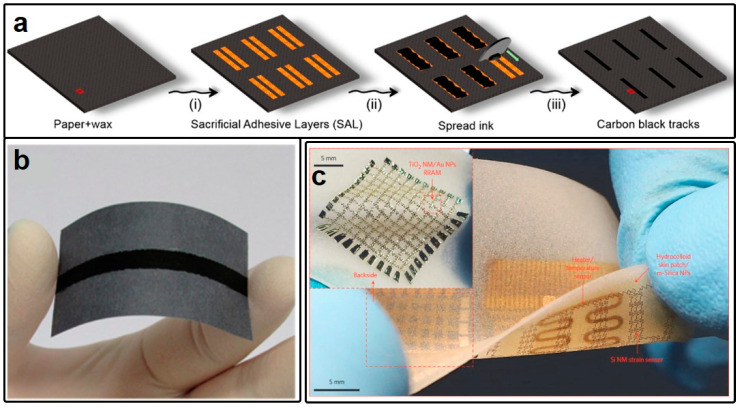
(**a**) Fabrication process of the paper-based graphene transmission line and the picture of the prototype in (**b**). (**c**) Picture of a wearable sensor system monitoring strain and temperature based on an elastomeric hydrocolloid patch. (**a**,**b**) are reprinted with permission from Ref. [102], Copyright 2017 American Chemical Society. (**c**) is reprinted with permission from Ref. [104], Copyright Springer Nature.

Another comparable soft substrate is conventional office paper, which has garnered significant attention in the domain of paper electronics due to its outstanding cost-effectiveness. Office paper has been widely employed in the design and fabrication of various applications, including energy harvesting [97,98,99] and RFID [100,101]. Nevertheless, due to its inability to withstand the high temperatures required for soldering, the creation of conductive layers on paper typically relies on inkjet printing techniques. For instance, as reported by Murilo Santhiago, regular office paper coated with wax serves as the substrate, while carbon black (CB) functions as the conductive ink [102]. By applying the conductive ink to a sacrificial adhesive layer and subsequently removing the layer, specific electrical patterns with predefined functions can be constructed, as illustrated in Figure 4a,b. Moreover, Yadong Xu et al. proposed in Ref. [103] the use of pencils for drawing specific circuit diagrams on paper, simplifying the manufacturing process and reducing fabrication costs. However, wearable circuits fabricated on either PI or paper substrates are limited in their ability to bend within a certain angle, and lack the flexibility required for stretch or twist manipulations, resulting in poor compatibility with the skin’s surface. To address these limitations and enhance twistability and stretchability, Donghee Son et al. have developed a multifunctional wearable device based on an elastomeric hydrocolloid skin patch, as depicted in Figure 4c [104]. This integrated wearable system involves a more intricate fabrication process in which the electronic system is initially prepared on a silicon substrate and then transferred to the skin patch. Although the fabrication process is complex, it yields a versatile device with a high level of integration.

Wearable antennas and functional circuits constitute two major components of wearable electronic systems, each with distinct requirements for fabrication. Nevertheless, a delicate balance must be made between low-cost manufacturing, ease of implementation, high efficacy, and exceptional flexibility. The pursuit of materials that can achieve such a compromise is of paramount importance in ensuring that wearable systems simultaneously exhibit all these desirable attributes.

## 3. Design of Wearable Electronics for Different Functions

Traditional copper-based electronic systems, such as antennas and sensors, typically operate with stable and desired performance characteristics when deployed in free space, owing to their favorable electrical properties. Conversely, wearable electronics confront challenges leading to reduced functionality, including diminished gain in wearable antennas and reduced sensitivity in wearable sensors, primarily attributable to the degraded electrical properties of both conductor and substrate materials, characterized by low conductivity and significant dielectric losses. Addressing these challenges necessitates the implementation of specific antenna and circuit designs to empower wearable systems with the requisite capabilities for diverse functions.

**Figure 5:**
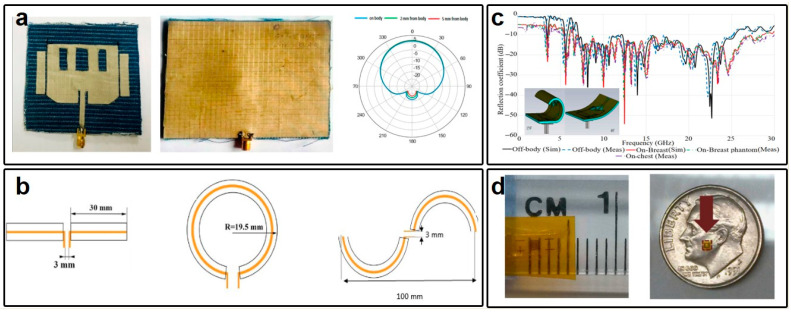
(**a**) Top and bottom views of a textile-based wearable antenna and its radiation patterns when placed at different distances from the human body. (**b**) Three different antenna specifications were used to examine the shift of operation frequency due to the operation in close proximity to the human body. (**c**) Top and bending views of the wearable antenna fabricated using PDMS and nickel–copper–silver. (**d**) Pictures of the implantable antenna for ICP measurement based on PI and copper. (**a**) is reprinted with permission from Ref. [105], Copyright 2020 Taylor & Francis. (**b**) and (**c**) are reprinted from open-access literature [106,107]. (**d**) is reprinted with permission from Ref. [108], Copyright 2014 Springer Nature.

One common issue that wearable antenna may encounter points to the frequency shift induced by their operation in proximity to the human body—an electromagnetic energy absorber. Consequently, their operation status may undergo significant degradation compared to free-space operations. As reported in Ref. [95], the operation frequency of nanomaterial-based wearable antennas strongly depends on the distance between the antenna and the human skin. Furthermore, since human tissues exhibit losses, a substantial portion of the radiated energy from the wearable antenna may be absorbed by the human body, leading to both degraded antenna performance and potential harm to humans. Addressing these challenges may be accomplished through two approaches. The first approach leverages the sensitivity of the wearable antenna’s operation frequency to the distance from the human skin for sensing purposes, as previously demonstrated in Ref. [95]. The second approach involves the use of grounded antenna designs. For example, Pranita Manish Potey and collaborators proposed a denim-based textile antenna with a grounded microstrip patch configuration (Figure 5a) [105]. This configuration results in a large main lobe-to-back lobe power ratio, with the majority of power directed away from the backside of the wearable antenna. Additionally, the operation frequency of the proposed wearable antennas remains nearly unchanged. In regard to the antenna specifications, Mourad Oudjane et al. proposed wearable antennas based on multilateral fibers for wireless communications (Figure 5b) [106]. Specifically, three types of antenna specifications, namely, loop, dipole, and spiral, were explored to illustrate the impact of the human body on antenna operation frequency. Their investigations reveal that the dipole antenna exhibited the most stable performance when considering the human phantom, while the loop antenna experienced the most pronounced frequency shift. This finding suggests that dipole and spiral antennas may be suitable for far-field wireless communication applications, ensuring relatively stable operation frequencies when worn on the body.

Additionally, broadband antennas with sufficient bandwidths to accommodate frequency shifts when placed close to the human body may also be desirable. As an illustration, Qammer H. Abbasi and his research group introduced an ultra-wideband (UWB) wearable antenna fabricated from conductive woven fabrics, shown in Figure 5c, operating within the bandwidth of 5 to 25 GHz, which is sufficiently wide to cover potential frequency shifts when the antenna is in close proximity to the human body [107]. Moving a step forward, in the realm of NFC, coil antennas represent a favorable choice for power and information transfer due to their reliance on magnetic couplings, which are less susceptible to interference from the human body. As demonstrated by Lisa Y. Chen et al. (Figure 5d), this coil antenna operates within the GHz range based on its size [108]. The authors successfully conducted in vivo measurements of intracranial pressure (ICP) in rats by implementing a coil antenna inside the rat’s skull. Particularly, the presence of animal tissue did not adversely affect the performance of the coil antennas, ensuring the success of the ICP measurement.

**Figure 6:**
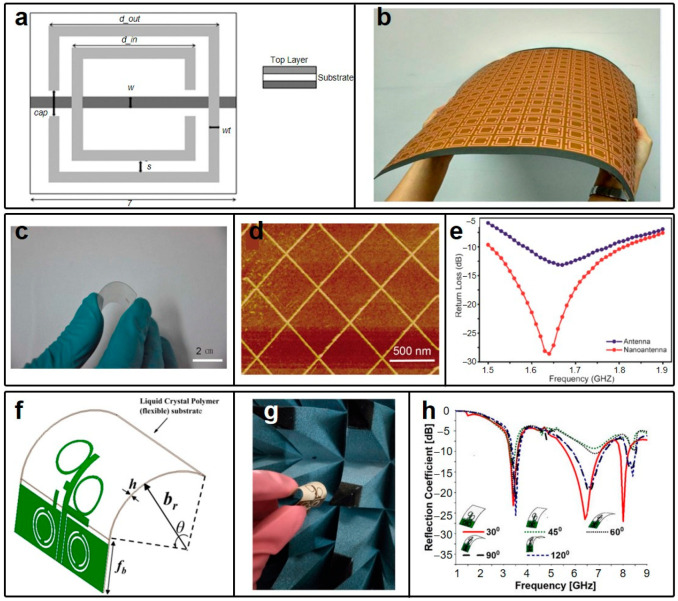
Schematic of the unit cell in (**a**) and picture of the overall device of the metamaterial-inspired antenna in Ref. [113]. (**c**) Photograph of the nanopatterned AU metamaterial antenna when subject to bending. (**d**) Atomic force microscope (AFM) obtained picture of the unit cells. (**e**) Operating frequency of the nanopatterned metamaterial antenna. Schematic (**f**) and picture (**g**) of the flexible metamaterial-based antenna in Ref. [115] when subject to bending. (**h**) Shift of operating frequency of the meta-antenna under different bending angles. (**a**,**b**) are reprinted from open-access literature [113]. (**c**–**e**) are reprinted with permission from Ref. [114], Copyright 2018 IOP Publishing. (**f**–**h**) are reprinted with permission from Ref. [115], Copyright 2019 John Wiley & Sons.

Recent years have seen a surge in research interest in metamaterials-inspired antennas, particularly within the realm of soft electronics. These antennas offer novel ways to manipulate incident electromagnetic waves, making them ideal for applications in impedance matching, sensing, and energy harvesting [109,110,111,112]. A notable example of such an antenna, detailed in Ref. [113], employs a split ring resonator (SRR) structure (Figure 6a), a common feature in metamaterial-based designs. This particular antenna operates at ~2.8 GHz. However, by adding a 0.3 mn-thick ferrite film atop the SRR, its operating frequency drops to ~2 GHz. Despite its flexibility, shown in Figure 6b, the antenna’s size is too large for practical use in body-area network applications.

To address the size issue, Dingxin Liu and colleagues have developed a compact, transparent nanopatterned Au antenna, as shown in Figure 6c [114]. This antenna’s flexibility, transparency, and small size make it an ideal candidate for integration with clothing and on-body scenarios. Unlike the previously discussed meta-antenna using traditional copper and FR4, the unit cell of this one is fabricated using electron beam evaporation on a PET substrate, which is shown in Figure 6d using an atomic force microscope (AFM). Surprisingly, this antenna can have nearly 35% efficiency around its operating frequency, 1.65 GHz (Figure 6e), which is sufficiently large for supporting communications in biomedical applications. This may indicate a potential future direction of metamaterial-based antennas, which is the combination of soft materials like PET with metamaterial-based antenna specifications.

However, the design of these antennas is still facing some challenges. For instance, the large size and number of unit cells make their practical implementation difficult (Ref. [113]), and they also have a high fabrication cost (Ref. [114]). An interesting solution can be seen in Figure 6f, proposed by Manikonda Venkateswara Rao et al. [115], where a circularly polarized metamaterial-inspired antenna is made with just four unit cells (Figure 6f) using copper and liquid crystal polymer (LCP). This compact antenna demonstrates significant bendability, as shown in Figure 6g, attributed to the great mechanical properties of the substrate. Further, the operating frequency of this antenna is plotted in Figure 6h under different bending angles, illustrating great robustness against such bending.

To summarize, the metamaterial-based antennas may exhibit reduced size, enhanced bandwidth, and improved gain and efficiency compared to the traditional antenna specifications, which may complement the performance reduction caused by the emerging flexible materials, making them particularly suitable for biomedical applications.

**Figure 7:**
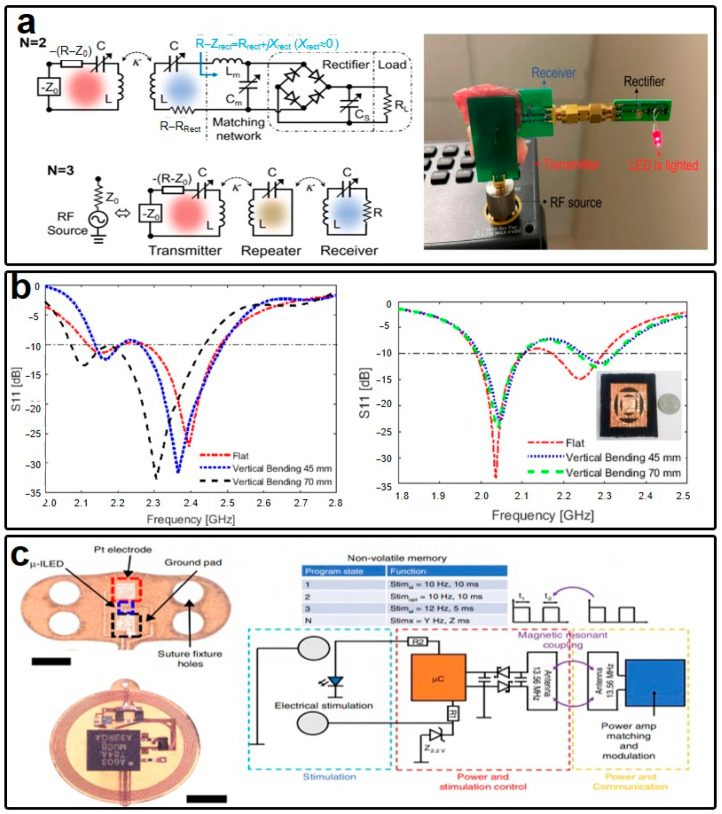
(**a**) Schematic of the robust wireless power transfer system for biomedical applications and its prototype. (**b**) The shift of operation frequencies of wearable antennas with respect to the deformation manipulations. (**c**) The shift of operation frequencies of wearable antennas with respect to the deformation manipulations with and without the nanomaterial coating. (**c**) The picture and system diagram of a wound healing system based on various chips and PI substrate. (**a**) is reprinted with permission from Ref. [116], Copyright 22022 IEEE. (**b**) is reprinted with permission from Ref. [118], Copyright 22023 Springer Nature. (**c**) is reprinted from open-access literature [119].

Beyond antenna specifications, an efficient method for maintaining a fixed operation frequency in electronic systems, such as on-body wireless power transfer, can be achieved through specific system designs. Zhilu Ye et al. (Figure 7a) theoretically and experimentally demonstrated a wireless power transfer system for bio-implantable applications employing a non-Hermitian structure [116]. Diverging from the conventional wireless power transfer system involving simple and direct coupling between two coil antennas, this non-Hermitian system incorporates specialized circuit designs on both the receiver and transmitter ends. This unique modification results in a power transfer system with a fixed operation frequency, independent of the presence of human tissues and other external influences, as validated through in vitro experimentation.

The operation frequency of wearable antennas can experience shifts not only when the antennas are placed directly on the human skin, but also due to deformations such as bending manipulations. A systematic investigation by Lingnan Song examined how bending manipulations affect the performance of rectangular patch antennas in wearable applications [117]. This study explored both E-plane and H-plane bending, and revealed significant shifts in resonance frequency and gain reduction resulting from E-plane bending. Notably, the frequency shift caused by the human body was found to be less pronounced than that induced by bending manipulations. This observation opens opportunities for utilizing such wearable antennas for sensing purposes. For instance, wearable antennas attached to joints could effectively monitor joint motions, as reflected in their resonance frequencies. Some specific antenna designs have been developed to mitigate the performance degradation caused by deformations in wearable antennas. Suman Pattnaik and his research group introduced a nanomaterial-coated denim-based wearable antenna that maintains a consistent operation frequency with and without bending manipulations [118]. In this design, a perovskite dielectric nanomaterial is coated on the denim substrate of the antenna. This nanomaterial coating effectively smooths the antenna’s surface and fills air gaps in the multilayer textile substrate, addressing the issue of frequency shifts induced by bending (Figure 7b).

In contrast, wearable circuits are less affected by the human body due to their relatively low-frequency operation. However, in turn, biocompatibility is of utmost importance in the design of wearable circuits. An exemplary case is the pacemaker, which operates in close proximity to the heart. John A. Rogers and his group proposed an implantable multimodal pacemaker powered wirelessly (Figure 7c) [119]. This wearable circuit consists of three units: stimulation, power management, and communication. We should point out that the power management and communication units are located outside the animal body, while the stimulation unit (Pt electrodes) is implanted inside. This arrangement ensures that chip-based wearable circuits do not disrupt the internal environment of the animal, while the electrodes directly attached to the heart are fully biocompatible. It is evident that chip-based wearable circuits, due to the presence of rigid electronic components, cannot be fully implanted. Therefore, the group further refined their design and materials, proposing a fully implantable and bioresorbable pacemaker in Ref. [84]. It is natural to conclude that chip-based wearable circuits may be more suitable for epidermal sensing scenarios requiring complex functions, as exemplified in Refs. [120,121], while implantable systems prefer a chipless schematic, as seen in Ref. [84]. A typical example of chip-based wearable circuits is a wound healing sensor, where wearable circuits are integrated into wound dressings with the assistance of other polymers. Under such conditions, the rigid chips may not directly interfere with the wound sites while fulfilling complex sensing functions. In contrast, chipless wearable circuits are typically preferred for low-power and single-mode sensing or power transfer scenarios. In summary, wearable antennas and circuits adopt different emphases depending on their functionalities, which, in turn, dictate their distinct design strategies. Nonetheless, the ultimate form of wearable electronics, characterized by being fully chipless, battery-free, wireless, and flexible, would minimize the influence on the human body while maximizing electronic capabilities.

## 4. Wearable Electronics-Based Biomedical Sensing

In the preceding section, we provided a concise overview of material selection and fabrication, along with a discussion of specifications and designs relevant to various wearable electronic devices, encompassing specific applications. In the subsequent section, we will center our attention on the practical applications of wearable antennas and circuits within the domain of biomedical sensing. It is noteworthy that biomedical sensing can be broadly categorized into two primary classes: epidermal sensors and implantable sensors. Here, we commence with an exploration of epidermal sensors.

**Figure 8:**
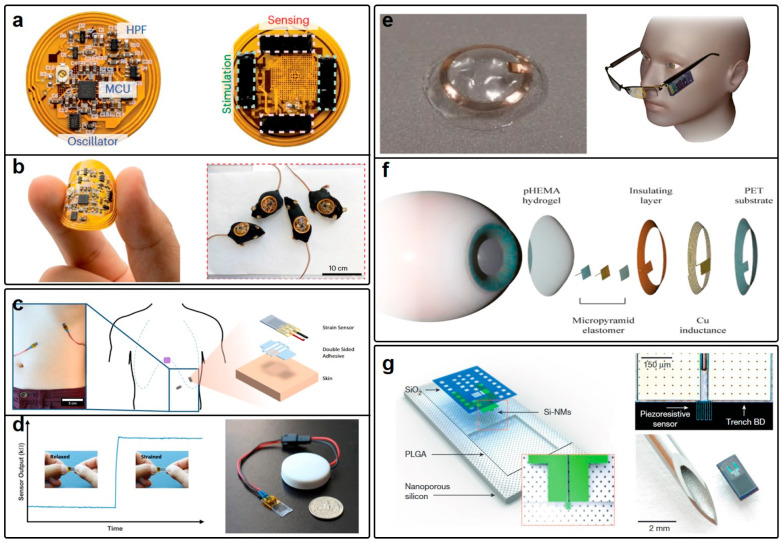
(**a**) Top and bottom views of a highly integrated wound healing sensor. (**b**) Pictures showing the flexibility and biocompatibility of the wound healing sensor in (**a**). (**c**) Sensing diagram and the structure of the wearable respiration rate monitoring sensor. (**d**) Sensing capability and data transmission system of the respiration rate monitoring sensor. (**e**) Close-up view of the wearable IOP sensor in the left panel and the entire sensing system with the readout device integrated with the glasses in the right panel. (**f**) The implementation of the IOP sensor combined with other polymer materials to ensure biocompatibility. (**g**) The structure of the implantable ICP sensor and the microscopic picture of the sensor. A size comparison is also included. (**a**,**b**) are reprinted with permission from Ref. [122], Copyright 2022 Springer Nature. (**c**,**d**) are reprinted from open-access literature [123]. (**e**,**f**) is reprinted with permission from Ref. [124], Copyright 22022 American Chemical Society. (**g**) is reprinted with permission from Ref. [125], Copyright 2016 Springer Nature.

At present, the most prevalent type of epidermal sensor is represented by wound healing sensors, often constructed using FPCB. These sensors have reached a significant degree of maturity in terms of potential industrialization, with Figure 8a showing one typical example. Interestingly, these wound healing sensors, due to their physical separation from the human skin facilitated by FPCB, have the capacity to support complex electronic systems comprising memory storage, control units, power management, Bluetooth connectivity, and sensing modules. The wound healing system depicted in Figure 8a was conceived by Zhenan Bao and his research group, and it notably incorporates a hydrogel biointerface [122]. This hydrogel interface offers attributes such as low impedance and tunable adhesion, thereby ensuring concurrent sensing and healing procedures. The tunable adhesion feature of the bio-interface is particularly instrumental in facilitating detachment following the healing process, effectively preventing secondary damage to the wound site. Moreover, the hydrogel interface also serves the dual purpose of acting as electrodes, endowing the system with the capability to deliver electrical stimulation to the wound sites to expedite the healing process. Despite its high level of integration and multimodal sensing principles validated through experimentation on mice, the flexibility of this wound healing sensor is constrained by the FPCB substrate and rigid chips, as shown in Figure 8b. The excessive bending of the device can potentially compromise the integrity of these chips. In contrast, the performance of the antenna remains robust against device deformation due to the digital data transmission facilitated by the Bluetooth module.

Another exemplary instance of epidermal sensors is wearable respiration rate monitoring, as proposed by Michael Chu et al. [123] and presented in Figure 8c. This respiration rate monitoring system employs a strain sensor based on a piezoresistive metal film encapsulated within a silicone elastomer substrate. Variation in strain levels leads to differing resistance values in the metal film. Figure 8c illustrates the attachment of this strain sensor to the chest to monitor the variation of resistance during the deformation of the chest (Figure 8d). However, it is noteworthy that the authors have chosen to utilize an independent Bluetooth module (right panel of Figure 8d) to illustrate the sensor’s efficacy rather than integrating it seamlessly within the device. This tethered approach may have the potential to negatively impact the sensor’s performance and wearability. Furthermore, the sensor is adept at accurately measuring respiration volume based on the varying resistance of the strain sensor. In particular, this epidermal sensor, in contrast to the smart bandage, is characterized by its light weight, compact form and ease of implementation. To obviate the need for external Bluetooth modules and chip-based components for data transmission, this wearable respiration sensor can be integrated with analog NFC systems, as exemplified in Ref. [122]. This adaptation may endow the system with chipless and fully flexible attributes, enabling the continuous real-time monitoring of respiration rate and volume. It is important to acknowledge that this compromise necessitates that the wearable sensor is not capable of simultaneous monitoring of multiple indicators.

In addition to the aforementioned epidermal sensors, there are also many other wearable sensors directly attached to the human skin for diverse sensing applications. Examples encompass, but are not limited to, diabetes foot pressure sensors, ECG sensors, and sweat sensors. Each of these epidermal sensors serves a vital role in routine self-health monitoring.

Implantable sensors designed for biomedical sensing constitute a significant asset in the realm of clinical diagnosis, offering distinct advantages over their epidermal counterparts, given their primary focus on vital physiological signals. A salient example of their clinical utility is exemplified by intraocular pressure (IOP) sensors, which play a crucial role in the management of glaucoma. Fei Xu and his research group have introduced an implantable IOP sensor that harnesses a synergistic combination of hydrogel and a flexible coil antenna, depicted in Figure 8e [124]. At the same time, the read-out system can be simply integrated with the glasses, establishing the NFC system. The sensor configuration comprises a fixed inductance component, realized through the coil antenna, which concurrently functions as the information transmitter, and a parallel-plate-based capacitor featuring a pyramid microstructure. Alterations in IOP lead to changes in the relative distance between these plates, resulting in variations in effective capacitance. Consequently, the resonance frequency of the IOP sensor undergoes a discernible shift, which can be precisely detected by external monitoring equipment. Particularly, as depicted in Figure 8f, the copper-based coil antenna may combine with other materials with high biocompatibility and then come into contact with the inner eyelid when the eyes are closed, potentially causing discomfort and inciting inflammations. Therefore, there exists a compelling impetus to substitute the conductive materials of this IOP sensor with emerging materials that offer heightened biocompatibility. In light of the requirement for implantation, the incorporation of rigid chips is inadvisable in this context. Furthermore, it is imperative to emphasize that in scenarios characterized by minimal device deformation, such as this, the paramount consideration is the biocompatibility of the system, with flexibility ranking as a secondary concern.

Conversely, the monitoring of intracranial pressure (ICP) necessitates a wireless solution, as the prevailing state-of-the-art ICP measurements are reliant on catheter-based procedures, which are inherently uncomfortable and fraught with risks. An implantable sensor system capable of wirelessly monitoring ICP can effectively alleviate this predicament. John A. Rogers and his research group have proposed an implantable sensor that not only monitors ICP, but also intracranial temperature (ICT), concurrently through wireless means [125]. The implant, illustrated in Figure 8g, is characterized by its chipless and bioresorbable nature, exhibiting exceptional biocompatibility. The total size of this implantable sensor is comparable to the tip of a needle, making it highly desirable to be embedded into the human body. Additionally, this chipless implant embodies dual-mode sensing capabilities, permitting the simultaneous detection of ICP and ICT, marking a substantial advancement compared to prior chipless sensors. Remarkably, the sensor obviates the need for secondary surgical procedures for removal, as it can be absorbed by the body’s tissues and subsequently metabolized. Nevertheless, it is essential to acknowledge that the coupling of ICP and ICT data necessitates intricate post-transmission data processing, which may entail an augmented total cost for the sensing system. Moreover, the data transmitter utilized in this study is robust, but further refinements could be considered through the integration of wearable antennas, such as those referenced in Ref. [116]. Beyond these exemplars, there are also additional implantable sensors tailored for monitoring vital signals, encompassing parameters like intestine/gastric pressure and intravesical pressure. These implantable sensors capable of precisely and continuously monitoring vital signals via wireless wearable sensors may undeniably augment clinical care and enhance post-surgery treatment protocols. To make a better demonstration, we have constructed Table 1 to straightforwardly compare the characteristics of the wearable antennas and circuits reviewed previously.

## 5. Future Directions

The field of wearable electronics has undergone substantial expansion in recent decades, concomitant with the rapid advancements in 5G technologies. The ultimate form of wearable devices is centered on achieving attributes such as light weight, cost-effectiveness, ease of manipulation, and minimal risk to the human body. However, contemporary state-of-the-art epidermal sensors, as exemplified by the instances reviewed earlier, are often encumbered by rigid chip-based components and batteries. These components, while enabling vital functionalities, impose limitations on the flexibility of wearable devices and constrain the range of plausible implementation scenarios. Consequently, there exists a compelling imperative to develop chipless wearable electronics that seamlessly integrate comprehensive RF capabilities, encompassing sensing, data transmission, and electrical stimulation, while being constructed from ultra-flexible materials. Recent research endeavors have also explored innovative avenues, such as the development of tattoo-like wearable sensors [126,127] and the direct fabrication of functional circuits on the epidermis [128,129], emblematic of this ongoing pursuit.

Conversely, implantable systems designed for biomedical applications remain in their nascent stages, offering relatively rudimentary functionalities owing to the absence of complete electronic systems. The realization of an effective cross-body and in-body wireless power transfer system becomes imperative to support the operation of certain electronic systems, including sensing and electrical stimulation, within the context of implantable biomedical devices. Hence, the exploration of emerging materials endowed with the triad of attributes—namely, high electrical conductivity, biocompatibility, and flexibility—assumes paramount significance for the prospective development of implantable electronics. It is our contention that the forthcoming advances in both material science and electrical engineering are poised to catalyze significant progress in the realms of wearable electronics and biomedical applications.

## Figures and Tables

**Table 1:** Summary of substrates and conductors used in various wearable electronic systems.

Ref.	Substrate	Conductor	Conductivity (S/m)	Flexibility	Biocompatibility	Application	Cost
[65]	Jeans	Copper	6.0 × 10^7^	Poor	Poor	−	Low
[66]	Ecoflex 00-30	Silver fabric	1.1 × 10^3^	High	High	Joint motion	Moderate
[69]	PDMS	AgNW/PDMS	8.1 × 10^5^	Moderate	Moderate	−	Moderate
[74]	PET	Graphene	4.2 × 10^5^	Moderate	Moderate	NFC	Low
[82]	Epidermis	Gold	1.9 × 10^6^	High	High	Touch/Temp. */Pressure	High
[83]	FR-4	Gold/SWNT	−	Poor	Poor	Plantar pressure	Low
[84]	PLGA	W/Mg	2.3 × 10^7^	High	High	Pacemaker	High
[91]	PDMS	NCS95R-CR	−	Moderate	Moderate	−	Low
[92]	PET	Silver nanoparticles	−	High	Moderate	−	Moderate
[95]	Porous SEBS	AgNW/PEDOT: PSS	−	High	High	Cough	Moderate
[96]	PI	Copper	6.0 × 10^7^	Poor	Moderate	Plantar pressure	Low
[102]	Paper	Carbon black	−	Moderate	Moderate	Human–machine interface	High
[104]	Elastomeric hydrocolloid patch	Gold	1.9 × 10^6^	High	High	Temp./Strain	High
[105]	Denim	Nickel–copper–nickel	3.5 × 10^7^	High	High	−	Low
[106]	Cotton	Silver–glass polymer	−	High	High	Breath	High
[107]	Denim	Shieldit	1.2 × 10^5^	Moderate	Moderate	−	Low
[108]	PI	Copper	6.0 × 10^7^	Moderate	Moderate	ICP	Moderate
[116]	PI	Copper	6.0 × 10^7^	Moderate	Moderate	Wound healing	Low
[117]	Cotton	Copper	6.0 × 10^7^	Moderate	High	−	Low
[118]	Denim	Copper	6.0 × 10^7^	Moderate	High	−	Low
[119]	Parylene	Copper	6.0 × 10^7^	High	High	Stimulation	Moderate
[122]	PI	Copper	6.0 × 10^7^	Moderate	Moderate	Wound healing	Moderate
[123]	Silicon elastomer	Piezoresistive metal	−	Moderate	Moderate	Strain	High
[124]	Hydrogel	Copper	6.0 × 10^7^	Poor	High	IOP	High
[125]	Nanoporous Silicon	Mo	1.9 × 10^7^	High	High	ICP/ICT	High

* Temp. represents temperature.

## Data Availability

Not applicable.
